# Atypical Presentation of Ramsay Hunt Syndrome in an Immunocompromised Patient: A Case Report

**DOI:** 10.7759/cureus.118584

**Published:** 2026-10-25

**Authors:** David J Rash, Owori Mangeni

**Affiliations:** 1 Physical Medicine and Rehabilitation, Mercy Medical Center, New York, USA; 2 Neurology, LewisGale Medical Center, Salem, USA

**Keywords:** herpetic neuralgia, neuralgia, ramsay hunt syndrome (rhs), varicella-zoster, vesicular rash

## Abstract

Ramsay Hunt syndrome (RHS), or herpes zoster oticus, is a rare neurological complication of varicella-zoster virus reactivation within the geniculate ganglion, leading to unilateral facial pain, facial paralysis, otalgia, sensorineural hearing loss, and a painful vesicular rash on the face or ear. Reactivation of the virus most often affects the vestibulocochlear (cranial nerve eight) and facial (cranial nerve seven) nerves in RHS. Early recognition of RHS is important as delays in treatment can lead to permanent facial paralysis, scarring, or irreversible sensorineural hearing loss. In this case, we examine an 84-year-old immunocompromised female patient whose diagnosis of RHS was delayed by multiple medical comorbidities and an atypical initial presentation.

## Introduction

Ramsay Hunt syndrome (RHS) is caused by a human alpha-herpesvirus called the varicella zoster virus (VZV), which is responsible for both varicella (chickenpox) and varicella zoster (shingles). The virus has an incubation period of 1-2 weeks, with viral replication initially taking place in the respiratory mucosa followed by dermatologic migration with a vesicular rash. Viral spread is thought to take place through infected T cells transporting the virus systemically to the skin. During the initial infection, virus particles propagate to the sensory nerve cell bodies in the ganglia via the dorsal root ganglion (DRG) [1,2]. The precise mechanism by which the viral particles migrate remains unclear; however, human DRG xenograft studies have shown that the satellite cells’ interactions with neurons develop neuron-satellite cell complexes that promote rapid infection of neuronal cell bodies within the DRG [2]. The virus can lie dormant in the DRG for many years before reactivating and subsequently leading to a vesicular rash along a specific dermatomal pathway corresponding with that DRG’s innervation. Reactivation of the VZV typically occurs during a period of immunosuppression. In the case of RHS, the viral reactivation occurs along the geniculate ganglion of the facial nerve with possible involvement of the vestibulocochlear nerve as well [3]. The facial nerve supplies motor innervation to the facial muscles, parasympathetic innervation to oral and lacrimal glands, and relays sensory input from the anterior two-thirds of the tongue. Viral reactivation along the facial nerve can lead to ipsilateral facial paralysis, ear pain, vesicles in the auditory canal or auricle, altered taste perception, tinnitus, hearing loss, vertigo, and nystagmus. Diagnosis of RHS is often clinical and is based on symptoms such as unilateral facial vesicular lesions, hard palate or tongue pain, and facial weakness. Other common symptoms include abnormal tearing and saliva production, unilateral hearing loss, rhinorrhea, diffuse unilateral neuralgia, and blurry vision. Skin lesions are not always present, and around 2-23% of cases of RHS may not have any vesicular lesions at all [3]. The gold standard diagnosis is polymerase chain reaction of skin or saliva samples, but this is rarely done in clinical practice [4,5]. Prompt diagnosis of RHS is important as delayed treatment can lead to increased severity of facial paralysis and more persistent deficits following treatment. Treatment has traditionally been defined by corticosteroids and antivirals such as acyclovir or valacyclovir.

## Case presentation

Our patient is an 84-year-old woman with a past medical history of hypertension, type two diabetes mellitus, and breast cancer status post radiation therapy and immunotherapy with Keytruda. She presented to the emergency department for a five-day history of dizziness and weakness. She stated that she had a spinning sensation with movement, which induced nausea and improved with rest. She denied recent falls or illnesses. Initially, the workup in the emergency department was focused on cardiac causes of her dizziness and weakness, which revealed an electrocardiogram with normal sinus rhythm and frequent premature ventricular contractions but no ST segment elevations or depressions. Physical exam in the emergency department reported the patient had mild right-sided auricular swelling and an enlarged right-sided cervical lymph node. Due to the patient’s history of recent immunotherapy treatment with Keytruda, the patient was started on intravenous (IV) cefepime and IV fluids for suspected perichondritis versus other localized infection. Interestingly, no leukocytosis was present on admission, with a white blood cell (WBC) count of only 7,240 WBCs/microliter (normal range 4,500-11,000 WBCs/microliter). A non-contrast computed tomography (CT) scan of her head was obtained and did not show any acute hemorrhage or large core infarct. Our patient was admitted for suspected perichondritis and vestibular ataxia.

Upon admission, our patient was started on meclizine for the nausea, and IV vancomycin was added to IV cefepime for broader antimicrobial coverage while blood cultures were pending. The patient then revealed that she was also experiencing tongue pain in addition to the dizziness. Physical examination of the tongue did not reveal any lesions. On day two of her hospitalization, it was noticed that the patient had mild facial asymmetry with right-sided facial weakness. Our patient also reported tearing in the right eye and worsening pain in her right ear. Physical exam revealed both right upper and lower facial weakness with lid lag and worsening edema in the right ear. The National Institute of Health Stroke Scale (NIHSS) score was 3 for unilateral complete facial paralysis. A magnetic resonance imaging scan of her brain was ordered to rule out a cerebrovascular accident and did not show any acute intracranial abnormalities or signs of a stroke.

On day three of our patient’s hospitalization, she continued to endorse worsening right otalgia, tongue burning, and right-sided facial weakness. Otolaryngology was consulted and evaluated our patient. Based on their exam, they were suspicious for RHS given our patient’s immunocompromised status, unilateral facial weakness, and otalgia. We started our patient on 60 mg oral prednisone daily and IV acyclovir 500 mg q8h. Infectious disease specialists were consulted and recommended discontinuation of antibiotics and continuation of antiviral medication with inpatient ophthalmology evaluation. Based on their recommendations, the antibiotics were discontinued. Ophthalmology was consulted for worsening right facial nerve dysfunction with inability to close the right eye. Their recommendations were to tape the affected eye closed with breaks for lubricating eye drops and close monitoring for visual changes. It became apparent that a vesicular rash was developing in the patient’s right auricle with crusting and scant clear discharge in the external acoustic meatus, as demonstrated in Figure 1. The neurology team was consulted and recommended adding gabapentin. Based on their recommendations, the patient was started on gabapentin 200 mg three times a day for neuralgia. After several more days of antiviral therapy and high-dose steroids, our patient began to have significant clinical improvement. The vesicular rash began to crust over and heal, which corresponded with improvement in the patient’s reported pain level. Her facial asymmetry became less noticeable, as did her lid lag, and she was able to adequately close her right eye to protect it from injury.

**Figure 1:**
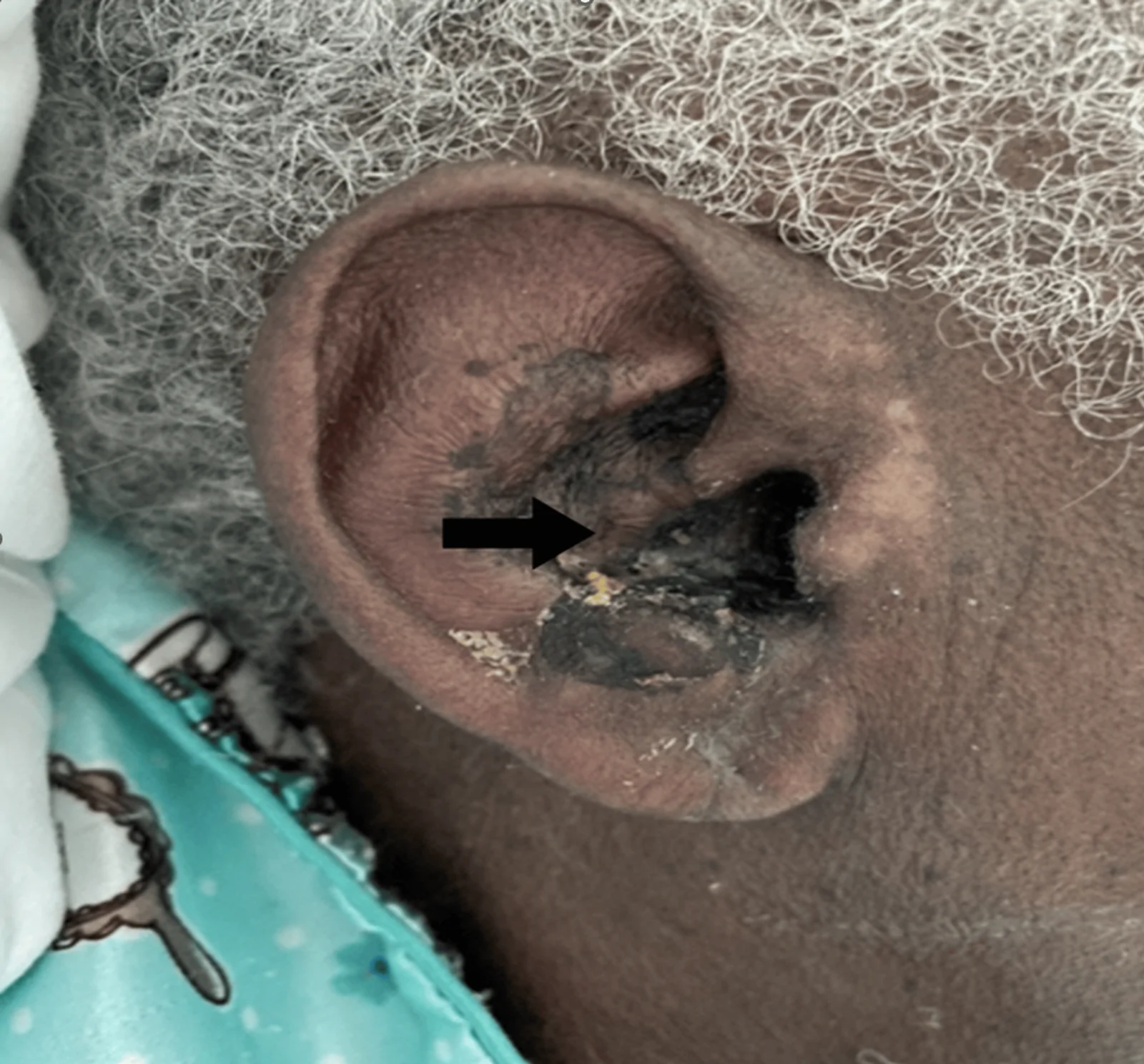
Resolving vesicular rash in the patient’s right ear highlighted by the arrow.

## Discussion

RHS is a complex neurological complication of VZV reactivation that requires prompt diagnosis, as delays in treatment can increase the risk of long-term facial nerve deficits. The differential for facial nerve palsy is vast and requires thorough investigation to distinguish between central and peripheral etiologies, as this may dictate medical management. Unilateral facial paralysis can be misdiagnosed as a forehead-sparing cortical infarct, a brainstem infarct, or can be attributed to Bell’s palsy, which can be caused by a wide range of conditions including viral infection, autoimmune disease, Lyme disease, or compression by malignant growth [6]. Sparing of the forehead musculature is often indicative that dysfunction is due to a cortical infarct, while weakness of forehead musculature indicates brainstem or peripheral nerve involvement. Unilateral ear pain is easily mistaken for otitis externa or perichondritis, and CT imaging may be of limited utility as findings are often nonspecific. Early development of the rash can be misclassified as contact dermatitis or cellulitis. It is therefore important for physicians to collaborate with otolaryngology, neurology, and ophthalmology in cases where RHS is on the differential. Failure to start corticosteroids and antivirals can lead to permanent facial paralysis and more severe scarring along the dermatomal segment [6,7]. Corneal damage can also occur due to exposure keratopathy if the affected eyelid isn’t properly addressed, which can lead to permanent visual impairment. Visual and auditory deficits can significantly impact cognition in our elderly patients as they may become more socially isolated and prone to accidents or falls. Facial weakness and dysphagia can lead to weight loss and failure to thrive, especially in the elderly population affected by RHS. In younger adults, the long-term complications of RHS can lead to worsening mental health related to scarring and residual facial nerve deficits. With prompt recognition and treatment, up to 70% of patients regain early facial nerve function [6]. Chronic neuralgia associated with delayed treatment can lead to insomnia, anxiety, and worsening of mood disorders in susceptible populations [8]. For all these reasons, providers must be cognizant of the signs and symptoms of RHS, especially in the elderly and immunocompromised populations.

## Conclusions

RHS is a rare disease that can present with vague, non-specific symptoms that can delay the diagnosis and treatment of this condition. In patients with symptoms concerning for RHS, it is essential that early treatment with antivirals and corticosteroids is initiated and that the patient is managed by a multidisciplinary team, as symptoms can cross many specialties. Additionally, it is important to follow these patients closely in the outpatient setting, as residual deficits and pain can have serious neurocognitive consequences, especially in our elderly populations. More research into multimodal pain management of persistent neuralgia from RHS is needed to optimize patient recovery.
